# Press for MIA production! The role of the Rho of plant GTPases in plant-specialized metabolism

**DOI:** 10.1093/plphys/kiae163

**Published:** 2024-03-14

**Authors:** Sara Selma

**Affiliations:** Assistant Features Editor, Plant Physiology, American Society of Plant Biologists; VIB Center for Plant Systems Biology, Ghent 9052, Belgium

Plants are the most sophisticated biofactories in nature, capable of synthesizing highly valuable compounds of industrial, pharmaceutical, or cosmetic interest. The tropical plant *Catharanthus roseus*, commonly known as Madagascar Periwinkle, is highly appreciated not only for its ornamental beauty but also for being a source of specialized metabolites with therapeutic properties called monoterpene indole alkaloids (MIA). Some examples include anticancer compounds, vinblastine and vincristine (Dhyani et al. 2022), or ajmalicine and serpentine, which are used in the treatment of hypertension (Kostin et al. 1986). The biosynthesis of MIAs is a highly intricate process involving over 50 enzymes from various pathways, transcription factors (TFs), intra-/intercellular signaling molecules, and transporters (Pan et al. 2016). Regulation of MIA biosynthesis is complex and likely involves post-translational modifications (PTMs) acting as molecular switches to modulate metabolic processes (Friso and van Wijk 2015). Geranylgeranylation is a PTM carried out by geranylgeranyltransferase (PGGT) that attaches a C20 geranylgeranyl lipid moiety to the target protein (Crowell and Huizinga 2009). Kumar et al. (2020) showed that both the silencing and overexpression of CrPGGT-I_β or geranylgeranyl diphosphate synthase 2 (CrGGPPS2), responsible for providing the geranylgeranyl moiety to CrPGGT-I_β, resulted in respective decreases and increases in MIA biosynthesis in *C. roseus*. These findings underscore the importance of geranylgeranylation in MIA production; however, the specific targets of protein geranylgeranylation and its role in modulating specialized metabolism remain unknown.

Some studies point to the Rho-of-plants (ROPs) proteins, a group of small plant-specific GTPases, as regulators of signaling that result in the modulation of specialized metabolism. ROP proteins act as molecular switches, undergoing conformational changes upon GTP binding and hydrolysis (Nielsen 2020). Additionally, it has been shown that the members of the Rho and Rac GTPases can be targets of geranylgeranylation, affecting different downstream responses (Huchelmann et al. 2014; Kurosaki and Taura 2015).

In this issue of Plant Physiology, Pedenla Bomzan et al. 2024 identify the ROP members in *C. roseus* (CrROP) that act as targets of protein geranylgeranyltransferases and are involved in the positive regulation of MIA biosynthesis.

As a first step, the authors classified the ROP members present in *C. roseus* by searching the Medicinal Plant Genomics Resource database. Six full-length coding sequences were identified (CrROP1-6). Phylogenetic analysis revealed that the identified CrROPs belong to 4 distinct groups, each associated with specific functions in plant development and signaling pathways (Fig. 1A). Interestingly, CrROP3 and CrROP5 (group 1) clustered together with SdRac2 and AmRac2, GTPases that positively regulated the production of specific alkaloids and sesquiterpenes, respectively (Asano et al. 2013; Kurosaki and Taura 2015). Additionally, the sequence alignment showed that all CrROPs possess characteristic G-box motifs and a hyper-variable C-terminal region. CrROP3, CrROP4, CrROP5, and CrROP6 were classified as type I ROPs, possessing a conserved potential CxxL geranylgeranylation motif at the C terminus. In the case of CrROP3 and CrROP5, they possessed the “CSIL” motif, while CrROP4 and CrROP6 contained the “CIFL” and “CILL” motifs, respectively. The authors used RT-qPCR to explore the spatial and temporal expression patterns of the identified CrROPs. All 6 *CrROPs* were found to be expressed in all the tissues, with *CrROP3* and *CrROP5* showing the highest overall expression in aerial parts, specifically in the leaves that are the production sites for certain MIAs.

**Figure 1.**
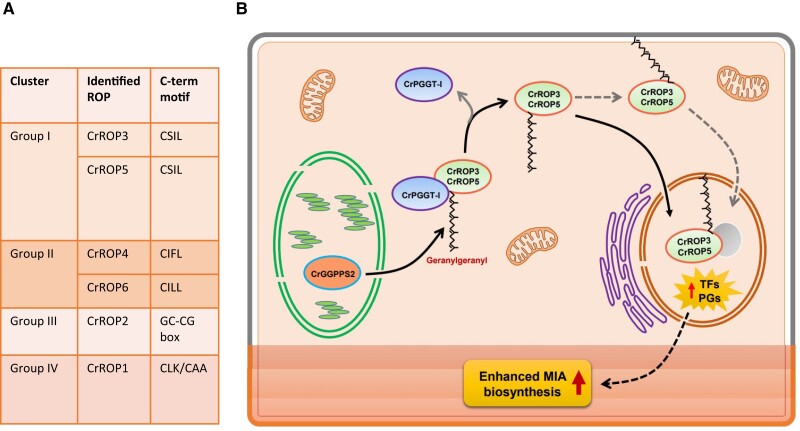
The role of ROP GTPases in the specialized metabolism. **A)** Identification and classification of the ROP proteins in *C. roseus*. **B)** Proposed model showing the role of CrROP3 and CrROP5 in the modulation of MIA accumulation in *C. roseus*. CrPGGT-I interacts with CrROP3 and CrROP5, and attaches a geranylgeranylate by utilizing GGPP made by plastidial CrGGPPS2. The geranylgeranylated CrROP3 and CrROP5 are either localized to the nucleus directly or indirectly via membrane anchoring and bring about transcriptional modulation of TFs and pathway genes (PGs) involved in MIA biosynthesis, which is localized in different cell types/layers of the leaf. The image was extracted form Pedenla Bomzan et al. 2024.

To experimentally confirm which CrROPs are geranylgeranylated, the authors performed an in vitro assay using purified CrROP proteins and fluorescent NBD-modified GGPP and PGGT-I protein. Interestingly, only the CrROP3 and CrROP5, characterized by the “CSIL” motif, were geranylgeranylated. This result matches with the previous results that show that CrROPs present a conserved CxxL motif at the C terminus that could be a target of geranylgeranylation. Additional yeast 2-hybrid assays supported the “CSIL” motif at the C terminus of CrROP3 and CrROP5 being crucial for the interaction with CrPGGT-I. Removing this motif abolished the interaction between CrROP3/CrROP5 and CrPGGT-I. Given these results, the authors determined the subcellular localization of the CrROP2, CrROP3, and CrROP5, expressing them as GFP fusion proteins (CrROP-GFP). The CrROP2 was localized in the plasma membrane, like most ROP GTPases (Chen et al. 2010), but, interestingly, CrROP3 and CrROP5 were exclusively localized in the nucleus. The authors suggest that the nuclear migration of CrROP3 and CrROP5 can be determined by the geranylgeranylation (Fig. 1B).

To investigate the role of *CrROP3* and *CrROP5* in MIA biosynthesis, their expression was downregulated in *C. roseus* leaves using virus-induced gene silencing. Downregulation of *CrROP2*, which is not geranylgeranylated, served as an additional control. The RT-qPCR analysis showed significant downregulation of transcript levels, without presenting visible phenotypes. The silencing of *CrROP3* and *CrROP5* triggered a notable reduction of the expression of the genes related to MIA biosynthesis, generating a significant decrease in secologanin and downstream MIA levels. In contrast, silencing *CrROP2* did not affect MIA biosynthesis genes or MIA amounts. In parallel, the transient overexpression of *CrROP3* and *CrROP5* significantly enhanced the expression of most MIA pathway genes and thus increased the accumulation of secologanin and MIA levels.

Finally, the authors assessed the role of the “CSIL” motif in the C terminus of CrROPs in the MIA biosynthesis, evaluating the overexpression of ΔCrROP3 and ΔCrROP5 mutants lacking the C-terminal “CSIL” motif. No effect on the accumulation of MIA in leaves overexpressing ΔCrROP3 and ΔCrROP5 was found, suggesting that the “CSIL” motif is crucial for the geranylgeranylation and the downstream responses in the MIA regulation.

In summary, the work provided by Pedenla Bomzan et al. proposes a new model where the geranylgeranylation can drive the localization of the ROP GTPases, CrROP3 and CrROP5, regulating the downstream signaling involved in the MIA biosynthesis. These findings describe a new mechanism of metabolic regulation and invite the identification of the downstream effectors of ROPs to obtain a complete image of the MIA-specialized metabolism in *C. roseus.*

## Dive Curated Terms

The following phenotypic, genotypic, and functional terms are of significance to the work described in this paper:

GTP CHEBI: CHEBI:15996

PTM AmiGo: PO:0005039

GGPP CHEBI: CHEBI:57533
